# Climatic variations and *Yersinia pestis* host-vector abundance: a case study in Ankazobe district to understand plague epidemiology in Madagascar

**DOI:** 10.1186/s12879-025-10929-z

**Published:** 2025-04-14

**Authors:** Fanohinjanaharinirina Rasoamalala, Henry G. Fell, Lanto A. Maminirina, Zaina Bodoarison, Lalatiana O. Randriamiharisoa, Mamy G. Randriamanantsoa, Haingotiana R. Ramambason, Voahangy Andrianaivoarimanana, Mireille Harimalala, Minoarisoa Rajerison, Beza Ramasindrazana, Steve Atkinson

**Affiliations:** 1 Plague Unit, Institut Pasteur de Madagascar, Antananarivo 101, PO. Box 1274, Ambatofotsikely, Madagascar; 2 University of Antananarivo, Antananarivo 101, PO. Box 566, Ankatso, Madagascar; 3 School of Geography, University of Nottingham, Nottingham, NG7 2RD UK; 4 Madagascar National Parks, Antananarivo 101, PO. Box 1424, Ambatobe, Madagascar; 5 National Plague Control Program, Ministry of Public Health, Antananarivo 101, PO. Box 88, Ambohidahy, Madagascar; 6 Health service and environment, Ministry of Public Health, Antananarivo 101, PO. Box 88, Ambohidahy, Madagascar; 7 The Biodiscovery Institute, School of Life Sciences, University of Nottingham, Nottingham, NG7 2RD UK; 8 Medical Entomology Unit, Institut Pasteur de Madagascar, Antananarivo 101, PO. Box 1274, Ambatofotsikely, Madagascar

**Keywords:** Plague, Fleas, Rodents, Climatic variables, Madagascar

## Abstract

**Background:**

Plague, a disease caused by the bacterium *Yersinia pestis* remains a major public health concern in Madagascar despite numerous multidisciplinary studies. The persistence of human plague infections is thought to be linked to fluctuations in mammalian host and flea populations, which are affected by climatic and environmental variations. This study explored local macro- and microclimatic variations along with mammal and flea population dynamics across different microhabitat types within plague endemic rural and forested habitats of Madagascar. Understanding these variables and their interdependent relationships may help us better understand the complexities of *Y. pestis* transmission in the Madagascan Highlands.

**Methods:**

Small mammals and their fleas were captured in different microhabitats within plague focus in the Ankazobe District of Madagascar. Simultaneously, climatic data including temperature and humidity, were collected to assess the potential relationship between flea population dynamics and climatic variations. Specialized equipment was used to monitor microclimate conditions across various microhabitat types and compare them with macroclimate. Monitoring was performed inside and outside rodent burrows located inside and outside houses and in adjacent forested areas.

**Results:**

A greater abundance of fleas was observed inside dwellings compared to other microhabitats, such as outside houses and forest, whereas small mammal species diversity was significantly higher in forest environments. We also revealed significant differences in microclimates across microhabitat types, with lower temperatures and higher humidity inside rodent burrows compared to outside burrows, outside houses and the forest. Inside houses, temperature variations were more stable although temperatures were higher and humidity lower inside rodent burrows compared to other microhabitats.

**Conclusion:**

This study highlights microclimate variation across different microhabitat types, which also differ from the macroclimate, and maps small mammal and flea abundance to these locations. These data suggest that it is important to further explore the relationship between microclimatic variations in the different microhabitats and the dynamics of flea and rodent populations as potential markers for plague persistence and transmission in these endemic foci.

**Supplementary Information:**

The online version contains supplementary material available at 10.1186/s12879-025-10929-z.

## Background

Plague, a re-emerging zoonotic and vector-borne disease, is caused by the Gram-negative bacterium *Yersinia pestis* [1]. This pathogen infects various vertebrate hosts, primarily small mammals [2], and is predominantly transmitted by fleas [1]. Human infection generally occurs following a bite from an infected flea which usually results in bubonic plague. Without treatment, the infection can progress to pulmonary or septicemic plague, both of which are associated with high mortality rates [3]. Between 2013 and 2018, the global mortality rate for plague was 17.5% among approximately 2,800 human cases, with 80% of these cases reported in Madagascar [4].

Three introduced small mammal species in Madagascar, *Rattus rattus*, *Rattus norvegicus* and *Suncus murinus* are known to serve as *Y. pestis* reservoirs [5–8]. Additionally, two flea species, the cosmopolitan flea *Xenopsylla cheopis* and an endemic flea species *Synopsyllus fonquerniei* are recognized as the main plague vectors [5–7]. These fleas and small mammals are found across various microhabitats including rural and forested habitats, which facilitates the spread and persistence of the disease [7, 9].

In Madagascar, plague outbreaks are primarily located in the Central Highlands at altitudes above 800 m [6] although some cases have been reported in the seaport of Mahajanga [8, 10, 11]. Outbreaks exhibit pronounced seasonality with high transmission rates during the warm and wet season from September to April [5, 6]. It is hypothesized that the Central Highlands provide favorable abiotic and biotic conditions for the maintenance and circulation of the disease by promoting the development and survival of small mammal and flea populations [12–14]. However, further research is needed to fully understand the influence of local climatic factors on the ecology of plague reservoirs and vectors.

Several studies have explored the relationship between flea population dynamics and climatic variables, suggesting their role in the seasonality of plague [15–19]. The two species of fleas in Madagascar exhibit distinct peaks in abundance and microhabitat preferences. *X. cheopis*, peaks around March and is mainly associated with small mammals captured inside houses. In contrast, *S. fonquerniei* peaks around November and is more frequently associated with small mammals caught outside houses and in forested habitats [6, 15, 16, 19]. Forested habitats could therefore play a role in maintaining plague by harboring reservoirs of small mammals and their fleas.

Previous studies have determined that climatic conditions influence the development of flea populations and their seasonal dynamics through both laboratory experiments and satellite-based climate analysis. For example, endemic *S. fonquerniei* was experimentally observed to have a lower thermal development threshold than *X. cheopis*. Despite the lower development threshold temperature, *S. fonquerniei* took significantly longer to develop across all temperatures tested (18–32 °C) while humidity (80-90% RH) did not have any appreciable effects on either species [16]. Flea abundance, intensity and infestation rates can be correlated with their location, either inside houses or outside with significant effects due to seasonality on outdoor-associated *S. fonquerniei*, but little effects for indoor-associated *X. cheopis* [15, 16, 19]. While laboratory studies can be extremely useful as they are easily controlled, the conditions may not accurately reflect the fluctuations observed in the field. Furthermore, the collection of satellite-derived climate data will not accurately reflect microclimates specific to certain microhabitats, particularly within burrows [16, 19].

Environmental conditions within burrows are known to be more stable than external conditions, however seasonal variation is still observed to a lesser degree within these microhabitats [16, 20]. *Y. pestis* has been sampled throughout village and forest environments in the rural highlands of Madagascar and hence the hosts, vectors and bacterium were exposed to varying microclimates between these habitats [6]. It is unknown how burrow microclimates may vary between these habitats, and to what degree this variation may impact host and vector abundance. In the burrow, vector species develop away from the host and are therefore wholly reliant on the burrow microclimate through much of their development [21]. We therefore hypothesize that variations in the burrow microclimate between habitats may lead to varying abundances and species composition of host and particularly vector species. Monitoring burrow conditions coincident with trapping and analyzing host and vector abundances, therefore represents the first steps to integrating microclimatic conditions into our understanding of plague dynamics in the Madagascan Highlands.

This study reports the variation in abundance of flea species based on host infestation rates, flea index and species diversity in relation to host abundance and diversity. We also explore the connection between these factors and climatic variations in microhabitats. Sampling locations included rodent burrows inside and outside rural houses as well as non-populated forested habitats. These data will help inform community healthcare strategies for the prediction and prevention of plague outbreaks as well as direct effective control strategies (insecticide and rodenticide, trapping, quarantine etc.) to affected regions in the event of cases being identified.

## Methods

### Study habitat and period

**Fig. 1:**
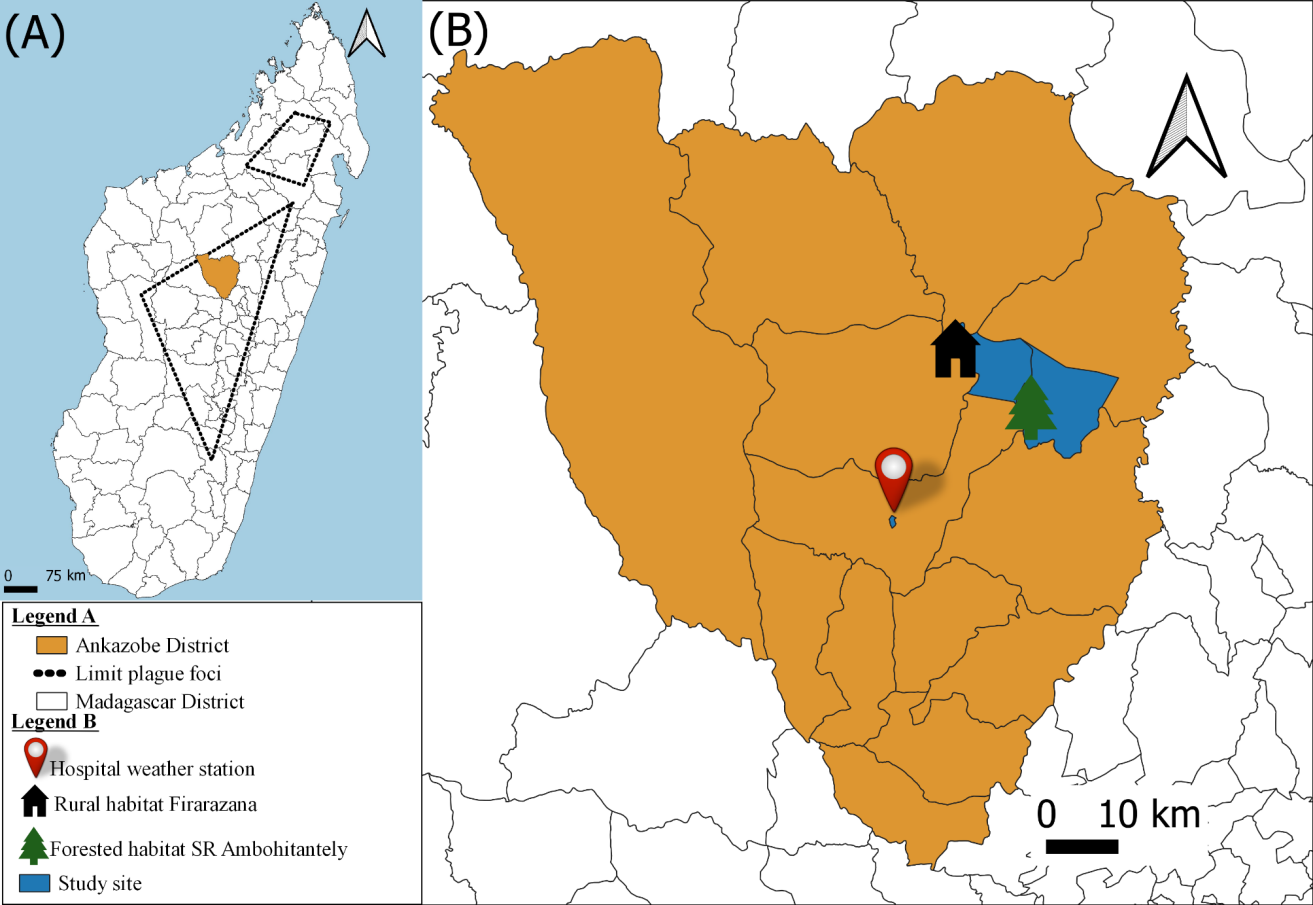
Map of the study site in Madagascar and the Ankazobe District. The black dashed line shows the plague endemic Highland regions of Madagascar and the location of the Ankazobe District (**A**) with and expanded map of the District showing the location of the study sites and weather station (**B**)

The capture of small mammals and climatic data collection were conducted between 4th to 12th April 2024 in the District of Ankazobe which is recognized as a focus for seasonal plague outbreaks (S 18°10’; E 47°46’; 1,200 m average elevation) (Fig. 1A). Two types of habitat were selected (1,527 m-1,600 m range elevation): the forested habitat of the Ambohitantely Special Reserve, consisting of a partially degraded montane rainforest, and the rural habitat of the village of Firarazana [9, 18, 19] (Fig. 1B). Throughout the manuscript, the term ‘habitat’ is used to designate large-scale rural or forested habitats and the term ‘microhabitat’ is used to represent small-scale local microhabitats inside a house, outside a house and in the forest.

### Sampling of small mammals and fleas

**Fig. 2:**
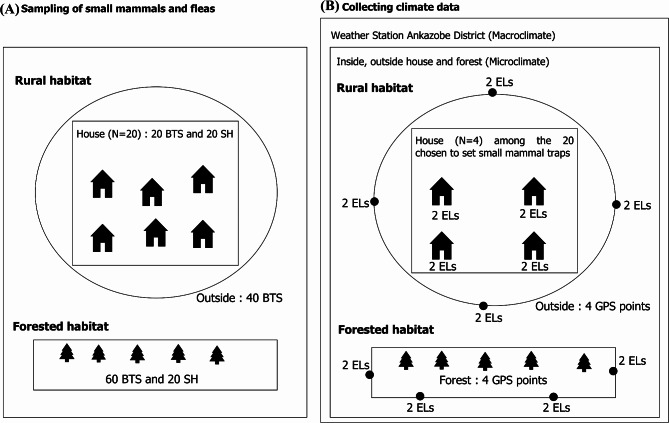
Methodology and locations used for capturing small mammals and collecting climatic data. (**A**) Methodology for capturing small mammals in different microhabitats. (**B**) Location of ELs in different microhabitats, including inside and outside burrows, inside and outside houses, and forest

Terrestrial small mammals were captured using wire mesh live traps (Besançon Technical Service (BTS), Besançon, France; 30 cm long × 10 cm wide × 10 cm high) and Sherman (SH) traps (H.B. Sherman Trap, Inc., Tallahassee, FL; 23 cm long × 7.5 cm wide × 9 cm high). Two different habitats (rural and forested) were selected for the sampling. In each habitat, 80 traps (60 BTS and 20 SH) were set and left in place for three nights of capture (Fig. 2A). The traps, baited with onion and dry fish, were laid out as follows: in the rural habitat, one BTS trap and one SH trap were placed inside each of the 20 randomly selected houses and the 40 BTS traps were set across a transect lines outside houses and spaced approximately 10 m apart. In the forested habitat, three BTS traps and one SH trap were set up along transect lines, spaced 10 m between traps, resulting in a total transect length of up to 800 m (Fig. 2A). The traps were checked twice daily, in the morning and in the afternoon and those containing animals were re-baited after the captured animals were removed. In the field, body measurements and morphological analysis of each specimen was assessed for species identification based on available guidelines [22]. All trapped endemic small mammal specimens were archieved for future studies. Fleas were collected by brushing the fur of the animal inside a 30 cm deep basin. Escaped fleas were collected using a home-made flea vacuum [23], which works by sucking up the fleas through a small tube and collecting them directly into a 20 cm glass tube. Fleas were preserved in 95% ethanol and then identified at species level in the laboratory using a low magnification binocular microscope and morphological keys [7, 24].

### Detection of *Yersinia pestis* in fleas

The detection of *Y. pestis* in fleas was carried out by testing all individual fleas collected from every small mammal captured. Fleas were ground in a Tissue Lyser II (Qiagen, Germany) containing two 3 mm tungsten beads (Qiagen, Germany), and DNA extracted using DNeasy Blood and Tissue kits (Qiagen, Germany) following the manufacturer’s protocol. DNA extracts were analyzed by quantitative PCR (qPCR) targeting the *Y. pestis* plasminogen activator (*pla*) gene (located on *Y. pestis*-specific pPst/Pcp1 plasmid) and the capsular antigen F1 (*caf1*) gene (located on the pFra plasmid) [1, 25, 26].

### Climate data

Microclimate data (temperature and relative humidity) were collected using ‘Easy Logger’ (EL) microclimate detection devices (Lascar Electronics, United Kingdom), which were installed at selected locations (Fig. 2B). Active rodent burrows were determined by the presence of droppings and once selected ELs were activated to measure temperature and humidity every 30 min over 24 h and placed 20 cm deep within the rodent burrows. At the rural habitat, eight ELs were placed inside the rodent burrows and outside four randomly selected houses that were chosen from the 20 houses used for trapping (two loggers *per* house) (Fig. 2B). Additionally, eight ELs were placed inside and outside (at a height of one meter) the rodent burrows along transect lines outside the houses, at a total of four GPS points representing four rodent burrows along the small mammal trapping transects (two loggers *per* GPS point) (Fig. 2B). In the forested habitat, eight ELs were placed along transect lines at four points (two loggers *per* GPS point) (Fig. 2B). The setup was the same for the forest and outside the houses of the rural habitat, with one EL installed in a rodent burrow and the other at a height of one meter near the rodent burrow location.

To measure the macroclimate, an ATMOS 41 weather station (METER Group, Inc., Pullman, WA, USA) was installed at the Ankazobe District Hospital Center to monitor climatic variations (1,269 m elevation). The station recorded climatic variations, including temperature and relative humidity. Measurements were taken every 30 min over a 24-hour period, corresponding to the days on which small mammals were collected in the study habitats.

**Fig. 4:**
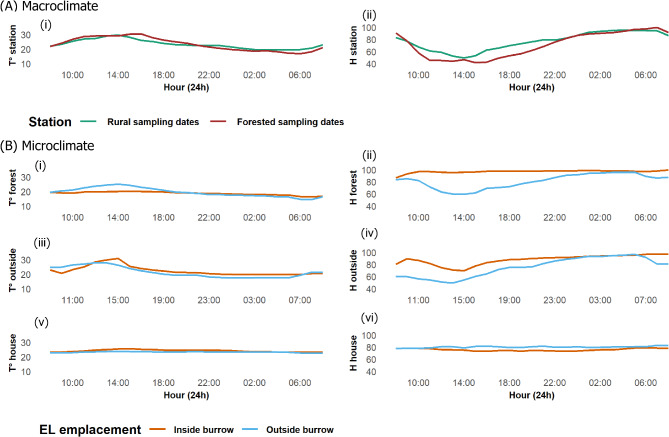
Illustration of the variation in temperature (T°) and humidity (H) between inside and outside the burrows in different microhabitats (inside/outside house and forest), together with the macroclimatic measurements recorded by the meteorological station. (**A**) Macroclimatic data showing the variations in temperature (**i**) and humidity (**ii**) between the two measurement dates. (**B**) Microclimatic data: variation in temperature and humidity in the forest (**i**; **ii**), outside house (**iii**; **iv**) and inside houses (**v**; **vi**)

Microclimate measurements were collected simultaneously with small mammal capture, from 8:30 a.m. on April 6 to 8:30 a.m. on April 7, 2024, in the forested habitat (forest), and from 8:30 a.m. on April 9 to 8:30 a.m. on April 10, 2024, in the rural habitats (inside and outside houses). These data were then compared to macroclimate measurement data from the weather station in Ankazobe across the corresponding sampling dates of the small mammal capture and microclimate monitoring in the rural habitat (“Rural sampling dates”) and the forested habitat (“Forested sampling dates”) (Fig. 4A).

### Statistical analysis

The statistical analyses were conducted based on small mammals collected over a three-night trapping period, with an equal number of traps placed in both rural and forested habitats. Each trap that successfully captured a small mammal represented a sampling point for each night of capture. For the climatic variation analysis, an equal number of data collection points were used for each location.

To assess flea abundance and species diversity, data were collected from trapping microhabitats inside houses, outside houses in rural habitats, and separately in forested habitats. Indicators of flea abundance were represented by the infestation rate and the flea index (Supplementary Table 1). The infestation rate was calculated as the ratio of small mammals infested with fleas to the total number of small mammals captured. The flea index was calculated as the total number of fleas collected out of the total number of small mammals captured [27]. Meanwhile, species diversity was quantified by the total number of species collected.

To examine the influence of microhabitat and host diversity on flea abundance and species diversity, we employed a generalized linear mixed-effects model (GLMM), implemented in the R package “*lme4*” [28]. The models incorporated fixed effects for microhabitat and host species (*R. rattus* or other), and a random intercept for the trap line, accounting for variability across trapping points. Since only 10% of the captured rodents were species other than *R. rattus*, these were grouped into a single category. Three distinct models were applied to analyze the impact of microhabitat and host species on flea abundance, specifically the infestation rate, flea index, and species diversity.

To analyze climatic variation across different location types (inside and outside burrows) and microhabitats (inside/outside houses and forest), we used also generalized linear mixed models (GLMM) with the “*lme4*” package [28]. The models incorporated fixed effects for microhabitat and a random intercept for GPS points to account for variability. The model compared the distributions of temperature and humidity (microclimate) as a function of the type of position of the EL (inside or outside the burrows) for each specific microhabitat (inside/outside house and forest). Additionally, it evaluated the overall differences in microclimate such as temperature and humidity between microhabitats (inside/outside house and forest) and in comparison, to macroclimate, taking into account the two types of position of the EL (inside or outside the burrows), in order to determine whether the variations observed were statistically significant, with a *p* threshold set at < 0.05. All statistical analyses were carried out using R version 4.3.0 [29]. These methods made it possible to take into account the variations between study sites to explore the impact of climatic factors on flea populations.

## Results

### Small mammals and flea sampling

A total of 111 individual small mammals representing five species were captured with *R. rattus* accounting for more than 90% of these captures (Table 1). In total, 38.7% of the mammals were trapped in the rural habitat (*N* = 43), either inside (55.8%; *N* = 24) or outside houses (44.2%; *N* = 19); while 61.3% of the small mammals were captured in the forested habitat (*N* = 68). Here *R. rattus* was found in both forested and rural habitats. *Nesogale dobsoni*, *Setifer setosus* and *S. murinus* were trapped in forested habitat whereas *Mus musculus* was found inside houses in the rural habitat.

**Table 1:** Number of small mammals and fleas in different microhabitats sampled

Locality	Small mammal species	Total number of small mammals	Total number of fleas	Small mammals infested by fleas	Infestation rate (%)	Flea index	Xenopsylla cheopis	Synopsyllus fonquerniei	Paractenopsyllus sp.	Echidnophaga gallinacea
Forest										
	*Nesogale dobsoni*	1								
	*Rattus rattus*	65	15	13	20.0%	0.2		14	1	
	*Setifer setosus*	1								
	*Suncus murinus*	1								
**Rural**										
**Outside house**										
	*Rattus rattus*	19	16	9	47.4%	0.8	16			
**Inside house**										
	*Mus musculus*	6	3	2	33.3%	0.5	3			
	*Rattus rattus*	18	78	11	61.1%	4.3	75	1		2
**Total**		**111**	**112**	**35**	**31.5%**	**1.0**	**94**	**15**	**1**	**2**

A total of 112 collected fleas represented four taxa, three of which were identified to species and one to genus level. These fleas included the two primary plague vectors, *X. cheopis* and *S. fonquerniei*, which were the most prevalent species in our sample (representing 84% and 13.4% of the total fleas, respectively) (Table 1).

### Flea abundance and small mammals diversity

**Fig. 3:**
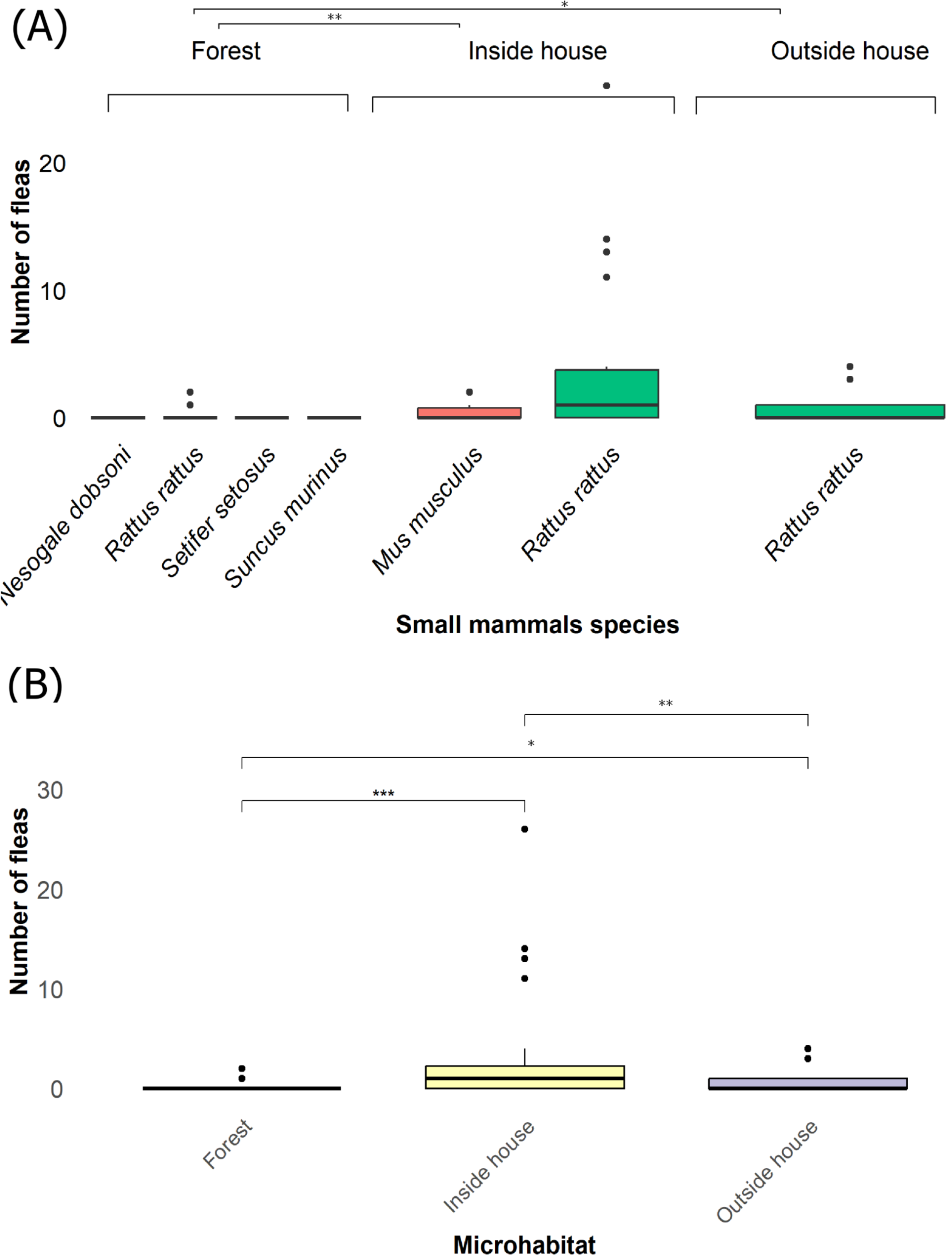
Results on small mammals and fleas sampling. (**A**) Illustrates the abundance of fleas collected from small mammals in different microhabitats (inside/outside houses and forest) and the results of statistical analyses of the infestation rate. (**B**) Shows the results of statistical analyses of the flea index, highlighting significant differences in flea abundance between microhabitats. (Significance codes: ‘***’ p = < 0.001, ‘**’ p = < 0.01, ‘*’ p = < 0.05 and ns = not significant)

The majority of *R. rattus* and *M. musculus* that were infested with fleas (primarily *X. cheopis*-Table 1), were collected inside houses with low numbers collected outside houses or forest (primarily *S. fonquerniei*-Table 1; Fig. 3A). Flea species diversity was not significantly different considering microhabitat (inside, outside house and forest) different habitats (rural and forested) (Supplementary Table 1). No flea species was found consistently across all microhabitats. *X. cheopis* was found only in the rural habitat and was completely absent from the forest. Comparatively, *S. fonquerniei* was most abundant in the forest and was sampled only once in the rural habitat (inside house). One *Paractenopsyllus sp.* was sampled in the forest and two *Echidnophaga gallinacea* were sampled in the rural habitat (inside house) (Table 1). Flea abundances, as indicated by infestation rates varied by microhabitat with the small mammals captured inside and outside houses showing significantly higher infestation compared to those in the forest (*p* < 0.01 and *p* < 0.05 respectively) (Fig. 3A) and between forested and rural habitat (*p* < 0.001) (Supplementary Table 1). Additionally, flea abundances measured by the flea index differed significantly between forested and rural habitats (*p* < 0.001) (Supplementary Table 1). The majority of fleas (86.6%, *N* = 97/112) were collected in rural habitats with *R. rattus* showing significantly higher flea counts compared to other small mammal species (*p* < 0.01). Flea indices were significantly different between the inside, outside of houses and forest (Fig. 3B). A significantly higher abundance of fleas was collected inside houses compared to those collected outside and in the forest (*p* < 0.01 and *p* < 0.001 respectively) (Fig. 3B). In addition, flea abundance was significantly higher outside houses compared to the forest (*p* < 0.05) (Fig. 3B). Furthermore, the flea index showed significantly higher flea counts in *R. rattus* compared to other small mammal species (*p* < 0.001) (Supplementary Table 1).

### Plague circulation in fleas

Of 112 fleas tested, none were positive for either *pla* or *caf1* (Supplementary Table 2) despite the sampling period falling within the high transmission period of plague in the Central Highlands of Madagascar [6].

### Climatic data analysis

Temperature and relative humidity were measured at 49 individual points covering the different microhabitats (Fig. 4A-B). Macroclimatic data showed minimal variation between the two measurement dates which corresponded to the periods when microclimatic variations were assessed in rural and forested habitats (Fig. 4A). Maximum temperatures and minimum humidity were consistently recorded around 2 p.m. (Fig. 4A). Microclimatic data revealed that in the forest, the temperature was lower and humidity was higher inside burrows compared to outside. Conversely, outside houses, burrow temperatures were slightly higher than ambient temperatures with increased humidity levels at night. Inside houses, temperature and humidity remained relatively stable, both inside and outside burrows (Fig. 4B). The temperature range for all microhabitats fell between 14 and 42 °C and the relative humidity between 40.9 and 100%. Maximum temperatures were recorded between 11 a.m. and 3 p.m., while maximum humidity was recorded at night (Fig. 4A-B).

### Comparison of climatic variations between the inside and outside of small mammal burrows according to each type of microhabitat

**Fig. 5:**
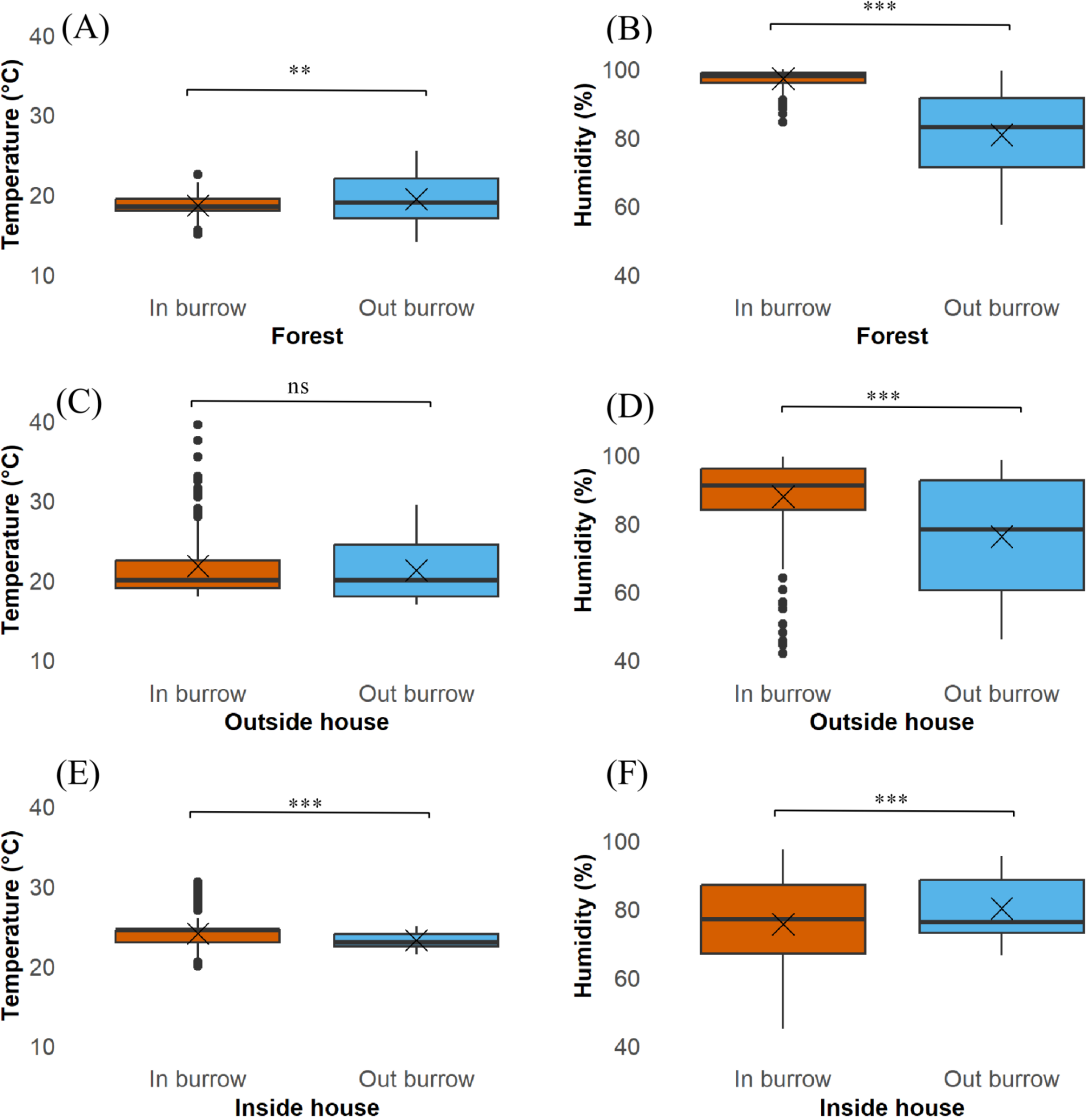
Climatic variations (temperature and humidity) between inside and outside of rodent burrows across different microhabitats, including forests (**A**, **B**), outside houses (**C**, **D**), and inside houses (**E**, **F**), analyzed using GLMM. Statistical significance is indicated as follows: ***p < 0.001, **p < 0.01, *p < 0.05 and ns = not significant

By analyzing the variation in climatic parameters inside and outside rodent burrows across different microhabitats (inside houses, outside houses, and forests), significant differences were observed (Fig. 5). In the forest, temperatures ranged from 15 °C to 22.5 °C inside burrows and from 14 °C to 25.5 °C outside the burrows (Fig. 5A). Humidity levels varied from 84.5 to 100% inside the burrows and from 54.5 to 99.5% outside (Fig. 5B). For the data collected outside houses, temperatures ranged from 18 °C to 42 °C inside rodent burrows, while outside burrows, they ranged from 17 °C to 29.5 °C (Fig. 5C). Humidity levels varied from 42 to 99.5% inside the burrows and from 46 to 98.5% outside (Fig. 5D). Inside houses, the temperature within burrows ranged from 20 °C to 30.5 °C, while burrows located outside had temperatures ranging from 21.5 °C to 25 °C (Fig. 5E). Humidity levels varied from 45 to 97.5% inside burrows, compared to 66.5–95.5% outside burrows (Fig. 5F).

Our GLMM analysis revealed that in the forest, temperature was significantly lower inside burrows compared to outside (*p* < 0.01) (Fig. 5A). Humidity also differed significantly between inside and outside the burrows (*p* < 0.001), being higher inside burrows with less variation (Fig. 5B). Outside houses, temperature was not significantly different inside burrows than outside (Fig. 5C). However, humidity was significantly higher inside burrows compared to outside (*p* < 0.001) (Fig. 5D). Inside houses, significant differences were found in both temperature (Fig. 5E) and humidity (Fig. 5F) between the inside and outside of rodent burrows (*p* < 0.001, respectively). The temperature was slightly higher inside the burrows, with greater variation in humidity, although humidity levels were slightly lower than those outside the burrows (Supplementary Table 1).

### Comparison of climatic variations between inside and outside house and forest

**Fig. 6:**
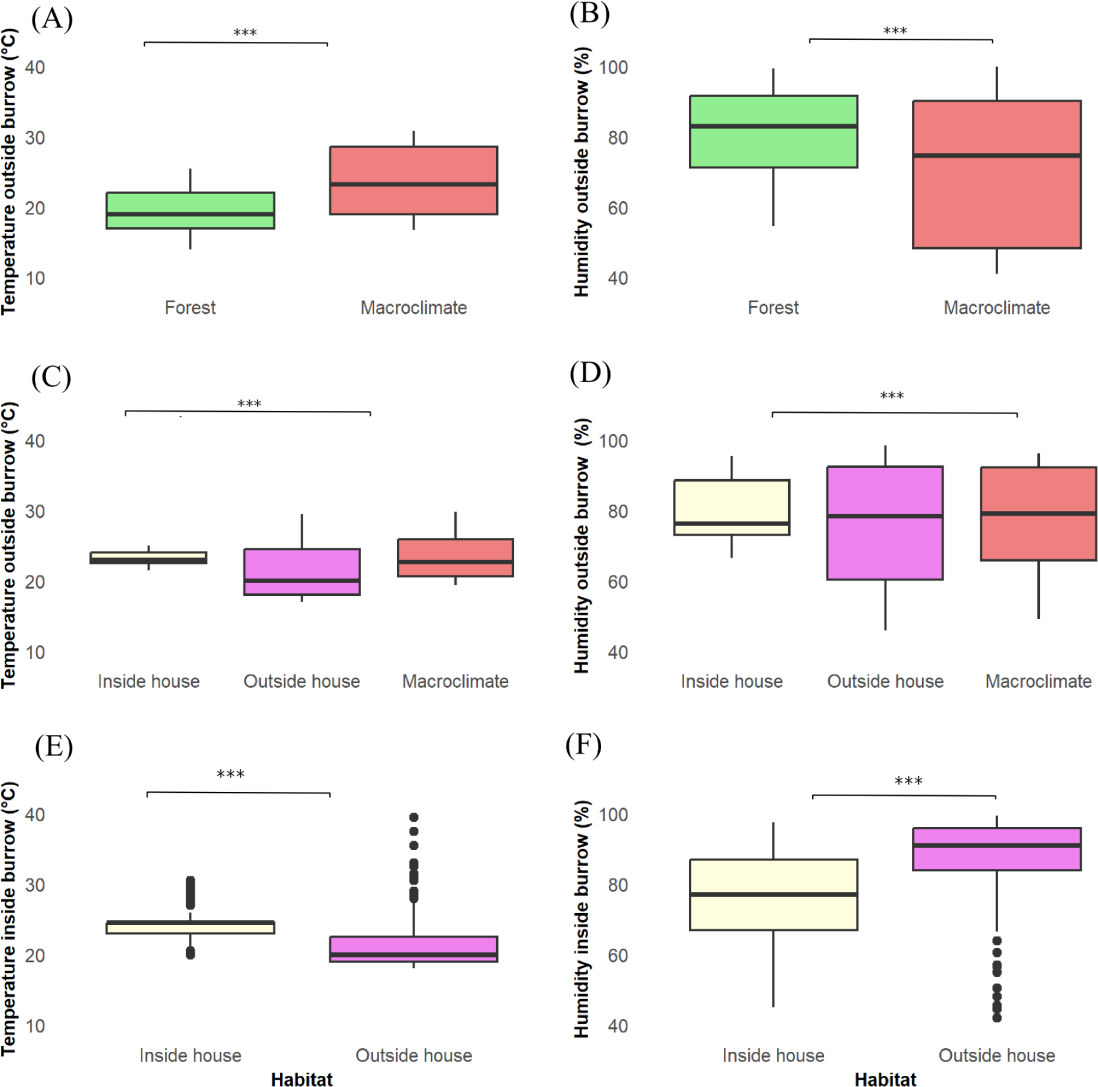
Comparison of climatic variations (temperature and humidity) across different microhabitats, including forested habitat and rural settings, using GLMM. Panels illustrate temperature (**A**, **C**, **E**) and humidity (**B**, **D**, **F**) variations between rodent burrows, inside/outside houses, forest and macroclimatic conditions. Statistical significance levels are indicated as follows: ***p < 0.001, **p < 0.01, *p < 0.05, ns = not significant

In the forest, a significant difference was noted between the climatic data outside the burrows and those at the climate station (Fig. 6A-B). The average temperature was lower (Fig. 6A) and relative humidity were higher in the forest (Fig. 6B) compared with the macroclimate measured at the station (*p* < 0.001 respectively). Outside the burrows in rural habitats, comparisons between inside and outside of houses (*p* < 0.001), as well as the macroclimate station, show a significant variation in temperature. The mean temperature inside the houses was significantly higher than outside the houses (*p* < 0.001) and no significant difference with the station’s macroclimate (Fig. 6C). Meanwhile, the temperature outside the houses was much lower compared to the macroclimate (*p* < 0.001). Similarly, humidity was significantly higher inside the houses than outside (*p* < 0.01), while the variation in the two microhabitats did not differ significantly in comparison to the macroclimate (Fig. 6D). There was noticeable temperature differences inside burrows located inside and outside houses (*p* < 0.001). The average temperature is higher and shows less variation inside houses than outside (Fig. 6E). Meanwhile, humidity inside houses is lower than outside (*p* < 0.001) (Fig. 6F) (Supplementary Table 1).

## Discussion

This study explored variations in the microclimatic conditions in different microhabitats such as rodent burrows inside and outside houses and forest, and compared them with macroclimatic data measured by a climate station installed in the Ankazobe District. Significant microclimatic variations were observed between microhabitats. At the same time, fluctuations in flea abundance, associated with the population dynamics of small terrestrial mammals in these microhabitats, were identified. These results suggest a potential influence of environmental and climatic variations on the population dynamics of flea vectors, which may ultimately impact on plague transmission in endemic foci in Madagascar.

To date, studies on the climatic conditions that influence *S. fonquerniei* and *X. cheopis* distribution and maintenance in Madagascar have been carried out in part by experimental laboratory studies or by measuring climatic variables at random locations from satellite-derived data [16, 19]. While these studies have provided valuable insights, there remains an opportunity to enhance our understanding of microclimates within specific microhabitats colonized by fleas and their small mammal hosts [15, 16, 19]. This study aimed to investigate microclimate variation across different microhabitat types and in addition, assess flea and small mammal distribution in these microhabitats. We hypothesize those microclimatic variations between microhabitats, which also differ from macroclimate at the district level, influence the distribution and abundance of small mammals and their fleas.

Our data supports previous studies reporting the abundance of *X. cheopis* [15, 16, 19], with over 84% of the insects collected, with high infestation rates and flea index inside houses compared to other species observed in this study. In fact, *X. cheopis* was observed on *R. rattus* collected inside houses but not in the forest. However, most of the *S. fonquerniei* individuals were observed in the forest as already supported by previous findings [7, 9, 30]. However, one individual of *S. fonquerniei* was observed inside a house. This was already observed during a two year surveillance in different communes in Ankazobe [19]. Flea abundance with rate infestation and flea index varied significantly across microhabitat types, such as inside or outside houses and in forest. Previous studies have highlighted the distinct climatic conditions required for the development of the two main plague vector species in Madagascar, which are found in different microhabitat types [16, 19]. *X. cheopis*, associated with small mammals living inside houses tolerates higher temperatures and humidity compared to *S. fonquerniei*, which is linked to small mammals in the outside environment and thrives in cooler habitats [5, 19, 31]. Obviously, each flea species tends to thrive in microhabitats that are most favorable to their development and consequently the variation in flea abundance may be attributed to differences in microclimate and environmental characteristics between these microhabitats.

A study carried out in the United States highlights the potential effect of environmental conditions, such as temperature and relative humidity, on the development of fleas in prairie dogs. The data was obtained using specific measurements of the microclimate in the burrows using iButton Hygrochron data loggers installed for four days and revealed that an increase in temperature and high relative humidity are associated with an increase in the flea population leading to high levels of plague transmission [20]. This underscores the critical importance of considering this variation when analyzing the relationship between climate and flea abundance. The distribution of flea species may be influenced by these specific climatic variations between microhabitats but further studies are necessary to confirm these observations.

Demonstrating the circulation of *Y. pestis* in fleas remains challenging, as highlighted by several studies that report low or even absent carriage rates of the bacterium [12, 13, 32]. However, evidence exists from previous research indicating that *Y. pestis* has been successfully isolated from fleas. For instance, a longitudinal surveillance study of flea collections from small mammals over two years in the same District of Ankazobe revealed a carriage rate of 0.02% (1 out of 5,295 fleas), with *X. cheopis* testing positive for *Y. pestis* [19]. Moreover, another study reported a carriage rate of 2% (4 out of 76 fleas tested) among two species, *E. gallinacea* and *X. cheopis* [33]. Additionally, research conducted in the Ambatofinandrahana District, an habitat recognized for its plague activity in Madagascar, indicated a carriage rate of 3.3% (9 positive individuals out of 274 tested). Interestingly, the flea species most commonly associated with humans, *Pulex irritans* tested positive, suggesting a potential role for this species in human-to-human transmission of plague in Madagascar [34]. The lack of positive flea samples in the present study must be considered within the context of sample size and location diversity, and the potential lack of bacterial circulation at the time of sampling, as no human plague cases were reported in the habitat during this period. Future research should aim to increase sampling efforts across various microhabitats to better assess the presence of *Y. pestis* in fleas. However, considering the extremely low number of *Y. pestis* positive flea samples across several studies, it is important to consider the role of fleas as the main source of infection into a human population at the beginning of a localized outbreak.

Regarding the climatic data, differences in elevation may not significantly influence our analysis of flea and host abundance or any potential relationship with climate variation. The primary focus of our climatic analysis is on variations between the inside and outside of rodent burrows across different microhabitats. The second level of analysis compares burrows inside and outside houses, and then external burrows across these microhabitats, alongside a comparison with the macroclimate of the district. Altitude remains relatively constant in the study sites, varying between 1,526 m and 1,600 m, while the climate station used to measure the macroclimate is located at 1,260 m. This small variation in altitude suggests that its influence on microclimatic variations is limited.

Furthermore, the temperature inside the burrows inside houses, shows variations with slightly higher temperature values recorded. This finding contrasts with earlier studies suggesting that burrows provide a more stable climatic environment with low temperature and high humidity [20], which is generally favorable for small mammals and the development of their fleas [15, 16, 35, 36]. However, such variation is not unexpected given that climatic conditions within burrows are undoubtedly influenced by many external factors including number and health of animals in the burrows, the size and number of nearby houses, type of crops, level of irrigation, degree of agricultural intervention etc [15, 20]. Temperatures inside the houses were higher compared to other microhabitats (outside houses and forest), although there was less variation in these conditions. This difference could be attributed to the protective structure of the houses, primarily composed of mud, with the roof made of metal sheets or plant materials which generate specific microclimates distinct from other types of housing. Further investigation is required to explore these aspects of microclimate variation both inside and outside rodent burrows in different types of houses.

We also show that the forest exhibited lower temperatures, higher humidity and a greater diversity of small mammal species. Human activity in rural habitats significantly disrupts natural microhabitats [22] compared to undisturbed forest and as a result, forested habitats offer better conditions. Higher biodiversity in forests may contribute to a dilution effect, where a greater diversity of host species reduces the risk of transmission of certain pathogens, including *Y. pestis*. Conversely, disturbed rural environments with lower biodiversity may favor the persistence of more competent hosts and vector fleas, which could increase the risk of disease transmission.

While our study does not quantitatively correlate flea abundance with climatic variations due to the constraints of a single sampling period and limited climate data collection, it highlights significant microclimatic differences among microhabitats. These findings suggest a potential relationship between microhabitat-specific climatic conditions and flea abundance, which may impact plague transmission dynamics. A longitudinal study with multiple trapping sessions over an extended period, alongside parallel climatic data collection, is necessary to establish a direct relationship between climate variability and flea abundance but was beyond the scope of this study. However, we do provide evidence of microclimatic differences across microhabitats and their potential association with flea abundance. Given that certain climatic conditions may favor flea development and proliferation, habitats with such conditions may be at a higher risk of plague transmission.

There is an urgent need for further investigations into the seasonal and annual climate and particularly microclimate variations and their impact on fleas and small mammal distribution. In addition, although the study highlighted significant differences between microhabitats, many other factors including human population density, agricultural practices and complex ecological interactions require urgent investigation. Unfortunately, we were unable to establish a definitive relationship between climatic variations and flea abundance across microhabitat types and habitats, as only one monitoring point was investigated in each study site. However, in terms of plague transmission dynamics, it is crucial to highlight the potential importance of forest dwelling small mammal species and their fleas in maintenance and persistence of plague. These species may be a conduit to *Y. pestis* transmission into surrounding rural regions when permissive environmental and climatic conditions permit, while also facilitating disease persistence among wildlife species.

In terms of flea control and plague prevention in Madagascar, this study may be a first step towards determining a relationship between host and vector abundance, microclimate variations and macroclimate at a local level. This information can help inform intervention strategies by identifying specific locations and optimal times to effectively reduce flea populations, thereby lowering the risk of plague transmission into the human population. For example, targeting specific burrows in particular microhabitats, whether inside or outside houses, which may provide favorable conditions for flea development, should be a priority. Measures such as improving housing conditions by eliminating burrows within dwellings will undoubtedly help create environments less conducive to flea survival and may also reduce proximity to rodents, thereby lowering the risk of flea-borne disease transmission. Additionally, continuous monitoring of climatic conditions and flea populations across different microhabitats would be crucial for anticipating and preventing plague outbreaks.

## Conclusion

The data presented here highlights significant variations in microclimates across different microhabitat types (inside/outside houses and forest) compared with the macroclimate in the Ankazobe District. These variations suggest a potential impact on flea population dynamics, particularly through differences in flea abundance between different types of microhabitats, which could potentially affect their development and survival conditions throughout the plague permissive season. This, in turn, may influence the dynamics of plague transmission. Continuous monitoring of climatic conditions, as well as rodent and flea populations across various local microhabitats, is essential for anticipating and preventing plague outbreaks. However, if fleas are not the main driver to initial infection, understanding more about what pushes the disease into the human population should be prioritized. An integrative and multidisciplinary approach, which considers both climatic variations and the ecological dynamics of reservoirs and vectors is therefore crucial for enhancing plague control and prevention strategies in the Central Highlands and, more broadly, throughout Madagascar. This would help preventing epidemics and improve public health in Madagascar.

## Electronic supplementary material

Below is the link to the electronic supplementary material.

Supplementary Material 1

Supplementary Material 2

## Data Availability

The datasets used and/or analyzed during the current study are available from the corresponding author on request.

## Abbreviations

BTS: Besançon Technical Service
EL: Easy Logger
PCR: Polymerase Chain Reaction
qPCR: Real-time Polymerase Chain Reaction
SH: Sherman (trap)
p: P-value
pla: Plasminogen Activator (gene)
caf1: Capsular Antigen F1 (gene)
