# Supplementary material for: Climatic variations and Yersinia pestis host-vector abundance: a case study in Ankazobe district to understand plague epidemiology in Madagascar
Source: BMC Infect Dis. 2025 Apr 14;25:521. doi: 10.1186/s12879-025-10929-z (PMC11995555; doi:10.1186/s12879-025-10929-z)
Supplement: Supplementary file 1 — Supplementary Material 1 [file 12879_2025_10929_MOESM1_ESM.docx]

**Supplementary Table 1. Parameter estimates from top models for factors influencing diversity species, infestation rate, flea index and climate based on Generalized Linear Mixed Models (GLMM)**

| **Analyses** | **Parameter** | **Estimate** | **SE** |
| --- | --- | --- | --- |
| **Diversity of flea species by habitat and microhabitat** | | |  |
| Intercept | Intercept | 6.9e-1^ns^ | 7.1e-1 |
| Habitat: rural vs forested | Rural | 2.7e-10^ns^ | 8.7e-1 |
| *Microhabitat* |  |  |  |
| Intercept | Intercept | 0.7^ns^ | 0.7 |
| Inside house vs forest | Inside house | 0.4^ns^ | 0.9 |
| Outside house vs forest | Outside house | -0.7^ns^ | 1.2 |
| Intercept | Intercept | 4.7^e^-11^ns^ | 1.0 |
| Inside vs outside house | Inside house | 1.1^ns^ | 1.2 |
| **Flea infestation rate by habitat and microhabitat** | | |  |
| Intercept | Intercept | -2.7*** | 0.01 |
| Habitat: rural vs forested | Rural | 1.7*** | 0.3 |
| Species *Rattus rattus* vs other species | Species *Rattus rattus* | 1.2*** | 0.01 |
| *Microhabitat* |  |  |  |
| Intercept | Intercept | -3.0* | 1.2 |
| Inside house vs forest | Inside house | 2** | 0.7 |
| Outside house vs forest | Outside house | 1.4* | 0.6 |
| Species *Rattus rattus* vs other species | Species *Rattus rattus* | 1.5^ns^ | 1.1 |
| Intercept | Intercept | -1.6^ns^ | 1.2 |
| Inside vs outside house | Inside house | 0.7^ns^ | 0.8 |
| **Flea index by habitat and microhabitat** | | |  |
| Intercept | Intercept | -4.7*** | 0.9 |
| Habitat: rural vs forested | Rural | 2.3*** | 0.5 |
| Species *Rattus rattus* vs other species | Species *Rattus rattus* | 2.4** | 0.8 |
| *Microhabitat* |  |  |  |
| Intercept | Intercept | -4.8*** | 0.9 |
| Inside house vs forest | Inside house | 3.0*** | 0.6 |
| Outside house vs forest | Outside house | 1.3* | 0.6 |
| Species *Rattus rattus* vs other species | Species *Rattus rattus* | 2.7*** | 0.8 |
| Intercept | Intercept | -3.5*** | 1.0 |
| Inside vs outside house | Inside house | 1.7** | 0.7 |
| **Climate analyze inside and outside burrow** |  |  |  |
| Intercept | Intercept | 3.2*** | 0.03 |
| Inside houses: inside vs outside burrow | Outside burrow temperature | - 0.04*** | 0.004 |
|  | Intercept | 4.3*** | 0.1 |
|  | Outside burrow humidity | 0.1*** | 0.01 |
| Intercept | Intercept | 3.1*** | 0.04 |
| Outside houses: inside vs outside burrow | Outside burrow temperature | -0.02 ^ns^ | 0.02 |
|  | Intercept | 4.5*** | 0.03 |
|  | Outside burrow humidity | -0.1*** | 0.02 |
| Intercept | Intercept | 2.9*** | 0.01 |
| Forest: inside vs outside burrow | Outside burrow temperature | 0.04** | 0.01 |
|  | Intercept | 4.6*** | 0.02 |
|  | Outside burrow humidity | -0.2*** | 0.01 |
| **Climate analyze between** **Microhabitats** |  |  |  |
| *Inside burrow: inside vs outside house* |  |  |  |
| Intercept | Intercept | 3.1*** | 0.01 |
| Inside burrow: inside vs outside house | Outside house temperature | -0.1*** | 0.02 |
|  | Intercept | 4.3*** | 0.01 |
|  | Outside house humidity | 0.2*** | 0.02 |
| *Outside burrow: inside vs outside house vs macroclimate* | |  |  |
| Intercept | Intercept | 3.1*** | 0.01 |
| Inside vs outside house temperature | Outside house temperature | -0.1*** | 0.01 |
| inside house vs macroclimate temperature | Macroclimate temperature | -0.01^ns^ | 0.02 |
| Intercept | Intercept | 3.1*** | 0.01 |
| Outside house vs macroclimate temperature | Macroclimate temperature | 0.1*** | 0.02 |
| Intercept | Intercept | 4.4*** | 0.01 |
| Inside vs outside house humidity | Outside house humidity | -0.1** | 0.02 |
| Inside house vs macroclimate humidity | Macroclimate humidity | -0.05^ns^ | 0.03 |
| Intercept | Intercept | 4.3*** | 0.01 |
| Outside house vs macroclimate humidity | Macroclimate humidity | 0.01^ns^ | 0.03 |
| *Outside burrow: Forest vs macroclimate* |  |  |  |
| Intercept | Intercept | 3.0*** | 0.01 |
| Outside burrow: forest vs macroclimate | Macroclimate temperature | 0.2*** | 0.03 |
|  | Intercept | 4.4*** | 0.01 |
|  | Macroclimate temperature | -0.1*** | 0.03 |

Statistical significance levels are indicated as follows: ***p < 0.001, **p < 0.01, *p < 0.05, ns = not significant.
