# Supplementary material for: Climatic variations and Yersinia pestis host-vector abundance: a case study in Ankazobe district to understand plague epidemiology in Madagascar
Source: BMC Infect Dis. 2025 Apr 14;25:521. doi: 10.1186/s12879-025-10929-z (PMC11995555; doi:10.1186/s12879-025-10929-z)
Supplement: Supplementary file 2 — Supplementary Material 2 [file 12879_2025_10929_MOESM2_ESM.docx]

| **Area** | **Habitat** | **Species fleas** | **Total Number of Fleas** | **PCR (Positive / Total Tested)** |
| --- | --- | --- | --- | --- |
| **Forest** | **Forest** | *Synopsyllus fonquerniei* | 14 | 0/14 |
|  |  | *Paractenopsyllus sp.* | 1 | 0/1 |
| **Rural** |  |  |  |  |
|  | **Outside house** | *Xenopsylla cheopis* | 16 | 0/16 |
|  |  |  |  |  |
|  | **Inside house** | *Xenopsylla cheopis* | 78 | 0/78 |
|  |  | *Synopsyllus fonqueriniei* | 1 | 0/1 |
|  |  | *Echidnophaga gallinacea* | 2 | 0/2 |

**Supplementary Table 2. Number of flea species in the various microhabitats sampled and results of molecular analyses.**
